# Reflections on agranular architecture: predictive coding in the motor cortex

**DOI:** 10.1016/j.tins.2013.09.004

**Published:** 2013-12

**Authors:** Stewart Shipp, Rick A. Adams, Karl J. Friston

**Affiliations:** 1 Department of Visual Neuroscience, UCL Institute of Ophthalmology, University College London, Bath Street, London, EC1V 9EL, UK; 2 The Wellcome Trust Centre for Neuroimaging, Institute of Neurology, University College London, Queen Square, London, WC1N 3BG, UK

## Abstract

The agranular architecture of motor cortex lacks a functional interpretation. Here, we consider a ‘predictive coding’ account of this unique feature based on asymmetries in hierarchical cortical connections. In sensory cortex, layer 4 (the granular layer) is the target of ascending pathways. We theorise that the operation of predictive coding in the motor system (a process termed ‘active inference’) provides a principled rationale for the apparent recession of the ascending pathway in motor cortex. The extension of this theory to interlaminar circuitry also accounts for a sub-class of ‘mirror neuron’ in motor cortex – whose activity is *suppressed* when observing an action –explaining how predictive coding can gate hierarchical processing to switch between perception and action.

## Highlights

• Predictive coding explains the recursive hierarchical structure of cortical processes.
• Granular layer 4, which relays ascending cortical pathways, is absent from motor cortex.
• Perceptual inference results if ascending sensory data modify sensory predictions action, if spinal reflexes enact descending motor and/or proprioceptive predictions.
• Motor layer 4 regresses as motor predictions inherently require less modification.

## Cortical architecture and hierarchical connectivity

Motor cortex was localised to the precentral gyrus of apes by Sherrington in 1901 [1], and was first identified histologically the following year by Campbell, using the brains of Sherrington's subjects [2]. Although Campbell emphasised the prominent fibre architecture of motor cortex, it is the cytoarchitectural tag ‘agranular’ cortex, coined by Brodmann [3] to describe his areas 4 and 6, that has proved the more enduring. Both authors used variations in cortical architecture for cartographic purposes, but these variants have rarely, if ever, been interpreted functionally; despite its ‘fame’, the dramatic recession of granular layer 4 in motor cortex has not attracted a single functional hypothesis.

### Sensory and sensorimotor hierarchies

Cortical layers are identified by cytoarchitecture, and further characterised by patterns of intrinsic axonal and dendritic arborisation [4]. Laminar distribution distinguishes consistent types of extrinsic corticocortical connection, classified as ascending, descending, and lateral [5]. These patterns are sufficiently conserved to identify a hierarchical organisation of areas in sensory systems (Box 1). Initial descriptions of sensorimotor hierarchies placed premotor above primary motor cortex (M1), with areas 3a and 3b (components of the primary somatosensory area, S1) at the lowest levels [5]. Our own survey aimed to establish the polarity of key reciprocal connections, but not to arrange areas into discrete tiers [6]. The absence of a distinct granular layer in primary motor cortex calls for some modification of the laminar criteria, but the presence of a cryptic layer 4 7, 8, 9 justifies the treatment of terminal patterns that target the layer 3/5 border zone as forward connections (or backward, if the pattern avoids this zone). Similar arguments apply to premotor cortex (Brodmann's area 6), sometimes described as ‘dysgranular’ [10], owing to a rudimentary granular layer.

Box 1 **Laminar specific connectivity and hierarchical distance**

Figure I shows the laminar sources and distributions of ascending connections (green) and descending connections (red, violet, and blue), originating from a certain level (i) in a hierarchical chain.

**Figure I:**
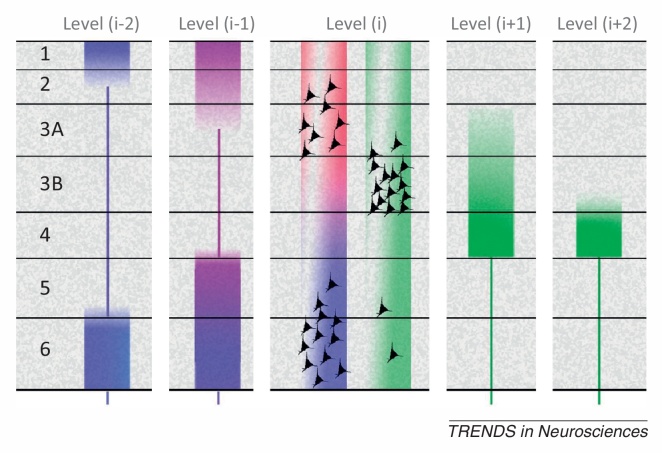
Prototypical laminar origins and terminations of connections in a hierarchy of sensory cortical areas (adapted from [4]).

The basic laminar patterns distinguishing ascending and descending connections were originally established by studies of primate visual cortex 5, 97, 98. Systematic variations with hierarchical distance were later formulated as a ‘distance rule’ 37, 99: with regard to origins, the proportion of superficial (layer 3A) neurons forming a backward projection decreases with greater distance spanned by the projection 99, 100, 101, 102, illustrated in Figure I by the ‘red’ terminals failing to contact level (i-2). The backward projection originating from deep layers reaches further, but these descending terminations (blue) show a progressive shift of focus upon layers 1 and 6 [51]. In the opposite direction, levels (i+1) and (i+2) show a progressive shift of focus of ascending terminations (green) upon layer 4 [97].

The differential contribution of superficial and deep sources to superficial and deep terminations in descending projections is not well established, because few studies have used tracers with subtotal layer deposition to study interareal connectivity. At minimum, the rule may be that like connects with like, laminar-wise. Layer 6, for instance, receives the densest input when the source of the descending projection includes layer 6 of the higher area 103, 104. However, layer 1 can receive descending input from deep layers in systems as diverse as primate visual and rodent somatomotor cortex 105, 106, and layer 5 can receive descending input from superficial sources, at least in cat and rat area V1 103, 104. These patterns are summarised in Figure I by the violet tone of descending terminations to layer 5 and superficial layers in level (i-1), indicating a mix of superficial (red) and deep (blue) sources at level (i). The blueing of terminals in deeper layers of level (i-1), and all layers in level (i-2), indicates a progressive domination of deep layer sources from level (i).

There is a consistent asymmetry between forward connections from sensory to motor areas and the reverse backward connections (e.g., between M1 and area 3a) 11, 12, 13, but the reciprocal connections among motor areas are of a distinct nature: there is a backward pattern of termination for projections from premotor areas to M1 [10], yet the reverse connections (e.g., M1 to SMA, supplementary motor area) are columnar 11, 12, 13, of the sort normally associated with lateral connections. Hence, the premotor areas may top the hierarchy, as previously suggested 5, 14, but there is little evidence for a classical ascending pathway through motor areas 6, 14. In this review, we attempt to reconcile the laminar architecture and connectivity in both visual and sensorimotor hierarchies within a popular theoretical framework for describing cortical operations [15].

## The principles of predictive coding

**Figure 1:**
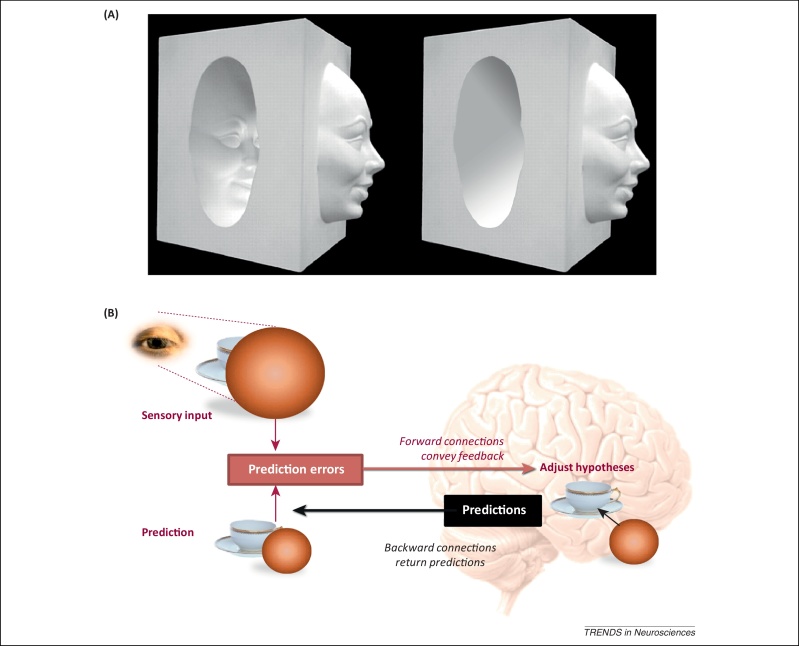
Predictive percepts and prediction error. **(A)** The hollow face illusion: on the left, the concave impression of a face is more readily seen as convex, lending it the appearance of a face mask, floating to one side of the vertical surface and illuminated from below. Once the facial features are removed (right), the cavity submits to the interpretation of being concave and illuminated from above. The illusion demonstrates the interplay of two prior expectations, concerning cause and context. The context is the attribution of a top-down light source: the featureless cavity continues to look concave, as if illuminated from above, even if the cue for a superior light source provided by the convex face is obscured. The cause is the attribution of a convex shape to an ovoid surface with facial features, which is sufficiently strong, in this illustration, to override the default contextual assumption of illumination from above. **(B)** Schematic illustrating the basic architecture of predictive coding. In this example, ambiguity (about occluded surfaces) is caused by visual occlusion that necessarily involves a nonlinear interaction between the causes of visual input (in that the appearance of an occluded object depends upon the occluder). This nonlinear perceptual inversion problem can only be resolved by a generative model that knows *a priori* that visual impressions are caused by objects. This problem is solved in predictive coding by generating descending predictions, given an initial hypothesis as to the cause, which is adjusted by ascending prediction errors. Adapted, with permission, from [107] (A).

A percept can be regarded as a hypothesis that explains sensory input 16, 17 – on occasion, an erroneous hypothesis, as demonstrated by classic illusions (Figure 1A). The percept interprets sensory data, such that what we see is the inferred cause of the sensations, not merely an image of the data *per se* [18]. In Figure 1A, the facial features have an ambiguous depth structure that is resolved by our past experience of convex faces. The ability to infer the cause of visual sensations (e.g., a face) rests on an internal, generative model of how objects generate sensory data 19, 20. Generative models are required to finesse the problem of sensory indeterminacy (e.g., ambiguity) that illusions aptly illustrate.

A generative model also has a temporal aspect: velocity is not a property of an instantaneous scene or ‘snapshot’, but an attribute that integrates sensory evidence over time. Biological motion detection implies recognition of complex motion patterns, such as a reach and grasp movement, or a repetitive action, such as walking [21]. In other words, the generative model of the brain is more like a narrative or scenario, predicting sequences of events. The scenario enables predictions about what may happen next. If a head is turning, for instance, a frontal view of a face may soon be replaced by a profile [22].

Generative models are necessarily hierarchical (in space and time). If the visual system operates as a generative model, the percept corresponding to a particular cause is not specified at only one level, but has multiple levels of description. Take face processing, for example: a high-level face area encodes view-invariant face identity, whereas lower levels are view specific but less identity specific [23]. Features such as hair, eye, and skin colour are also encoded elsewhere [24]. In addition, because face cells are size and position invariant [25], lower areas must represent the ‘filled-in surface’ and ‘border ownership’ attributes of a percept 26, 27, 28. In short, the gestalt of a ‘face’ has multiple components. In modelling terms, the high-level face area provides the highest stamp of recognition, guiding and contextualising inference about physical attributes in lower-level areas. Here, we shall use the term ‘expectations’ to refer to the representations of causes encoded at each level.

Predictive coding schemes (e.g., [29]) describe the inversion of a generative model, in order to recognise causes from their sensory consequences. In global terms, the model generates predictions of sensory input from high-level representations of causes; more specifically, the expectations at any given level predict the expectations at the level below. The model is inverted using a ‘guess it and try it’ approach (Figure 1B): each level computes ‘prediction errors’ by subtracting top-down predictions from its current expectations. The requisite predictions are based on expectations from the level above and conveyed by top-down or backward connections. Bottom-up prediction error signals are then passed forwards to modify expectations in the level above. This iterative, reciprocal exchange of predictions and errors minimises prediction error at every level of the hierarchy and provides a plausible explanation for visual sensations, in terms of expectations at multiple levels.

### Expectations: causes, states, and precisions

In generalised formulations of hierarchical predictive coding, there are three sorts of expectation: expected ‘causes’, ‘states’, and ‘precisions’ [15]. Causes are invariant aspects of the world that create regularities in sensory data, such as objects in the visual scene. Their correspondence to elements of the scene is concrete at lower levels (e.g., a colour), and increasingly abstract at higher levels of the hierarchy (e.g., a smile). Whereas causes model categorical aspects of the world, states model their dynamics; that is, the fluctuations caused by the interactions among causes (e.g., motion of an object) or between cause and context (e.g., a rotating object and its illumination). Finally, precision corresponds to the reliability (inverse amplitude of random fluctuations) of causes and states. Therefore, expected precision determines the relative confidence in descending predictions and ascending prediction error.

**Figure 2:**
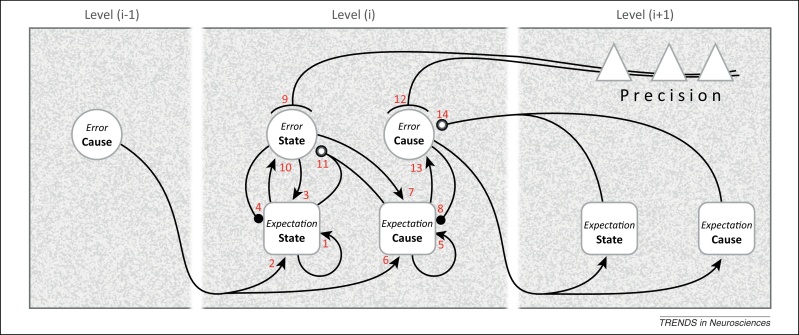
Graphical representation of the computational interactions between expectation and error units: the interactions depicted here are based on the differential equations describing the neuronal dynamics implied by generalised predictive coding (e.g., Equation 3 in [30]). Note the hierarchical structure: predictive coding involves recursive interactions among an arbitrary number of hierarchical levels, of which just one, level (i), is shown in full here. There are separate expectation and error units for causes and states (for definitions, see main text). The computations relating to causes and states are formally identical, except that the updates for causes are based on reciprocal exchanges between levels. In this scheme, expectation units recursively update their activity (1 and 5) with input from error units associated with other expectations (2, 3, 6, and 7), and predictions about themselves (4 and 8). The error units compare the activity of their associated expectation (10 and 13) with predictions based on a nonlinear function of other expectations (11 and 14); note that, for causes, this is a comparison of the expectation arising from the same level (13) with a prediction descending from the higher level (14). Crucially, the gain of error units is modulated by precision signals (9 and 12), shown here to originate from the higher level where they are regulated by expectations about causes and states, so rendering precision (i.e., gain control) context or state dependent. The relation with neural architecture is given in Figure 3, Figure 4. As portrayed here, the different computational units represent multilaminar neuronal ensembles: expectation units are square, error units are circular, and units mediating neuromodulation or precision are triangular. Connections with closed arrowheads are excitatory; connections with closed balls are inhibitory and linear; connections with open balls are inhibitory and nonlinear; and connections with arcs have a modulatory (gain) effect.

The differential equations describing predictive coding are provided elsewhere [30], together with the theory relating predictive coding to Bayesian inference 15, 31. Here, we consider the computational architecture and its implementation by neuronal circuitry. Figure 2 shows five kinds of computational unit (*cf*. neuronal ensembles): expectation and error units for causes and states, and units signalling expected precision. To recap, expectation units encode the expected causes and states describing events (scenarios) in the environment, whereas error units report inconsistencies between expectations at different levels or, at the sensory level, the mismatch between predictions and sensory input. Units encoding expected precision modulate the gain of error units and endow them with greater or lesser weight. This cortical gain control balances the influence of prediction errors at different levels in the hierarchy. Accordingly, precision is associated with the top-down deployment of attention [32] in the sensory domain and action selection in the context of affordance. In summary, expectation and error units interact to update beliefs about causes and states in the world, with one crucial distinction: expected causes are updated by reciprocal exchanges between hierarchical levels, whereas expected states are updated within each level.

Below, we suggest a neural implementation of the predictive coding model outlined above (noting that alternative formulations could specify a different neural architecture [33]). We prefabricate the scheme in visual cortex, as a model of hierarchical processing, before transcribing it to motor cortex and illustrating its explanatory scope through the example of mirror neurons.

## Neural implementation of predictive coding: sensory (visual) pathways

**Figure 3:**
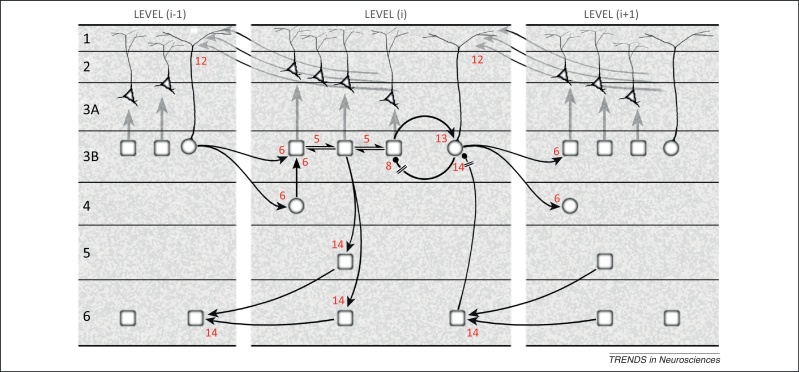
Neuronal interpretation of the message passing in Figure 2: the schematic illustrates the updating of expected causes, using extrinsic and intrinsic circuitry. The extrinsic connections are hierarchical and involve reciprocal connections of one area, level (i), with the levels above and below (Box 1). The intrinsic connections form a canonical microcircuit that has been summarised as a relay from layers 4 to 3 (and 2), and 3 to 5, with layer 6 receiving a certain level of input from all the layers above it 36, 108, 109, 110, 111. Conventions for the identity of computational units and labelled connections follow Figure 2, with the added proviso that all connections shown here arise from pyramidal cells; two connections (8 and 14) are explicitly indicated to require inhibitory relays, but all the remaining connections are likely to subsume contacts on both pyramidal neurons and interneurons. The circuitry may be summarised as follows. Forward, extrinsic connections terminate in layers 4 and 3B. The major output of layer 4 is to the superficial layers, where subclasses of pyramidal neurons represent expectations (rectangular units) and prediction errors (circular), or mediate precision (triangular). Activity in expectation units is maintained by recurrent interactions between pyramidal neurons and is informed by prediction error signals: excitation by the ascending prediction error arriving from layer 4, and inhibition from local error units in layers 2 and 3, which are the source of ascending prediction error for the level above. These superficial error units represent a subtraction of two signals: an excitatory signal received from their associated expectation units and a descending prediction, relayed by a local interneuron (see main text for further details). Interlaminar connections from layer 3 to layers 5 and 6 are likely to transmit all three classes of signal, but only the relay of expectation signals is shown here. These contact the deep pyramidal neurons that are the source of descending predictions. Precision signals arise from pyramidal neurons in layers 2 and 3A conditioned by input from expectation units, and form a descending chain of transmission through the superficial layers; these signals are capable of modulating error units via their apical tufts in layer 1. Overall, the scheme compiles anatomical data across species and visual areas. To provide but one example, it matches the particulars of local circuitry established for the relay of lateral geniculate nucleus (LGN) parvocellular signals through macaque V1 [42]: superficial expectation units can be associated with a class of intrinsic pyramidal neuron in layer 3B characterised by a short, tuftless apical dendrite, shown to receive direct input from the parvocellular input layer 4Cb; by contrast, layer 3B pyramidal neurons that have extrinsic axons always lack direct input from layer 4Cb, and express apical dendritic tufts in layer 1, characteristics associated with error units. This is consistent with a trisynaptic input–output architecture for the ascending parvocellular pathway, and the regulation of error units by descending precision signals.

We now attempt to marry the computational anatomy of predictive coding with cortical microcircuitry. For simplicity, we focus on updating expected causes: our aim is not to specify exactly how such computations are performed at the synaptic level, but to indicate how they might map onto the laminar architecture of extrinsic and intrinsic cortical connections. The scheme shown in Figure 3 is inferred from anatomy alone; there is no explicit physiological categorisation of the notional expectation, error and precision units, but we make the provisional assumption that all three are represented in some form by pyramidal cells (or by excitatory, spiny stellate cells in layer 4). Extrinsic and intrinsic axonal ramifications typically contact inhibitory interneurons as well as pyramidal neurons [34], but the former are largely excluded: for this reason, and others, we emphasise that Figure 3 presents a much simplified subset of known circuitry.

The scheme for updating expected causes (Figure 3) makes the fundamental assumption that ascending connections forward prediction error and descending (backward) connections convey predictions 19, 35, 36, 37. Prediction error is forwarded to level ‘*i*’ from superficial pyramidal cells of the level below (connection 6). As indicated in Box 1, the ascending axons typically terminate in both layer 4 and layer 3B 38, 39. The layer 4 cells retain the status of error units; they perhaps preprocess the error signals before relaying them to layer 3B; one such possibility is described in [40]. The cells in layer 3B receiving these inputs are deemed to be expectation units; note that expectation units have previously been assigned to the deep layers (5 and 6), where they act as the source of descending predictions 19, 35. Layer 6 receives collaterals of ascending axons carrying prediction error (not shown) 38, 39, 41, but the ascending projection targets mainly the superficial layers; therefore, expectation units must also exist superficially [36]. The superficial expectation units may correspond to a class of pyramidal cell lacking extrinsic output 42, 43. These units update and maintain expectations by collating ascending error signals, and by participating in recurrent interactions with each other (connection 5, representing the horizontal network of connections among pyramidal cells 44, 45, 46). Finally, expectation units enjoy a looped connection with specific error units in the same (3B) layer, excitatory in the forward direction but inhibitory in the backward direction, to which we must assign an intervening interneuron (connections 13 and 8).

In this scheme, layer 3B error units represent the point of convergence of the superficial and deep components of backward pathways, mediating precision and prediction, respectively. Precision exerts a modulatory or gating influence over error units that is compatible with nonlinear synaptic effects on apical dendritic arborisations in layer 1 (connection 12). The mechanism (as derived for large pyramidal neurons in layer 5 47, 48) involves back propagation of action potentials into the apical dendrite, lowering the threshold for apical calcium spiking and rendering the cell ‘exquisitely sensitive’ to backward input arriving at the apical tuft [49]. Hence, layer 3B error units should be pyramidal neurons that are modulated by descending precision signals via their apical tufts in layer 1, after initial (forward) activation by superficial expectation units. Other targets of superficial backward projections may include the apical tufts of pyramidal neurons in layers 3A and 2, themselves encoding precision, mediating a descending chain of precision signals through the superficial layers. Because the level of expected precision depends upon expected states of the world, precision signals are regulated by superficial expectation units through local axon collaterals. This same population of expectation units must also give rise to the deep component of the backward pathway, acting via an intrinsic relay to the source expectation units in layers 5 and 6 (connection 14).

This deeper descending stream generates predictions that act subtractively on error units in the level below. In our computational jargon, a ‘prediction’ is generated from an ‘expectation’ through a nonlinear transformation (involving backward connections; Figure 2). The generation of predictions is necessarily nonlinear, because it must model nonlinear interactions between the causes of sensory data (e.g., one object occluding another; Figure 1B). Furthermore, because one cause can have many consequences, a single expectation may generate separate predictions in different contexts and modalities. Neuronally, this reflects the fact that descending axons typically innervate several areas 6, 50, 51. Because these projections arise from pyramidal neurons in the higher area, and are excitatory, there has to be an intervening inhibitory neuron somewhere in the circuit. Although not outlawing alternatives, we have shown this pathway to operate through descending input to layer 6 pyramidal neurons, which transmit to superficial error units via layer 3 interneurons. This element of the proposed circuitry owes much to recent findings in mouse V1, where layer 6 pyramidal neurons were selectively activated *in vivo* with optogenetics [52]. This activation reduced visually driven activity in all pyramidal neurons recorded in layers 2–5. By contrast, inhibitory interneurons in layers 2–5 exhibited enhanced activity, suggesting a disynaptic suppressive influence of layer 6 on superficial layers: the relay we propose to layer 3B would be one element of this circuitry. Other studies of rodent V1 have shown that most inhibition in the superficial layers is generated locally, and have identified a class of interneuron receiving input from the deep layers 53, 54. This could be a somatostatin-positive (e.g., Martinotti) interneuron, known to exert subtractive inhibition 55, 56. Other mechanisms for the subtraction of predictions from expectations could involve direct contacts on superficial interneurons by axons of the backward projection. In layer 1, the effects can be modulatory and disinhibitory [57], but layer 2 harbours a greater concentration of interneurons [58], many of uncertain function.

Concluding with the deep layers, we depict intrinsic and extrinsic connections of layer 6 restricted to the reception and emission of descending expectation signals. Layer 5 not only shares this role, but may also transmit expected precision to lower areas, because it contributes to descending projections with superficial terminations (not shown, but see Box 1). Precision may be a crucial aspect of layer 5 function in motor cortex, considered below. Figure 3 omits one further subset of extrinsic connections: the ascending output from the deep layers. Note that these originate in a distinct subpopulation of cells in layers 5 and 6, not from cells with axons bifurcating to both ascending and descending targets [37]. We interpret this class as error units, and anticipate that they should include a complement of larger layer 5 pyramidal neurons with apical tufts in layer 1, as reasoned for superficial error units. Error units in layer 6 could be regulated by precision signals in layer 5. An example is the layer 6 ‘Meynert’ cell of primate V1, which has a short apical dendrite restricted to layer 5, and an ascending projection to area V5/MT [59]; it also has subcortical output to the colliculus 60, 61, contributing to a tecto-pulvinocortical loop 62, 63, but, importantly, is not among the corticogeniculate neurons of layer 6 64, 65.

### Corticothalamic feedback

The final link in the descending chain of predictions is from layer 6 of V1 to the lateral geniculate nucleus (LGN). The LGN may lack the sophisticated architecture of cortex, but can still be regarded as computing an error signal, by subtracting a cortical prediction from retinal input. The action of cortical feedback upon the LGN has been studied more intensively than any other descending pathway 66, 67. We note three findings in particular: (i) that corticogeniculate pyramidal cells lack apical dendrites ascending to layer 1 [68]; (ii) that most corticogeniculate neurons have receptive fields of ‘simple’ (rather than ‘complex’) organisation 69, 70; and (iii) that there is a direct phase reversal in the registration of cortical simple ON and OFF subfields, in relation to the OFF centre and ON surround (or vice versa) of the LGN relay neurons that they contact [71]. This arrangement precisely meets the requirements for producing a prediction error in the LGN [72].

## Neural implementation of predictive coding: motor pathways

We now apply the principles of predictive coding to the motor system. Expectations encoded in motor cortex predict the sensory state of the body consequent to action, predicting the proprioceptive dynamics of the movement trajectory (not only its final state) [73]. The obvious difference between perceptual (exteroceptive) predictions and active (proprioceptive) predictions is that, whereas the former change to accommodate sensory prediction errors, the latter can be fulfilled directly by classical reflex arcs. In this process, which we term ‘active inference’, proprioceptive predictions serve as motor intentions. Descending signals in motor pathways are traditionally referred to as ‘commands’, but ‘intentions’ is probably more apt, given that precise muscular forces are not prescribed, but organised by spinal reflex arcs in receipt of predictive signals. This issue and others (such as the relation of active inference to ‘optimal control’ theories of motor function) are discussed in greater detail elsewhere 6, 73, 74.

The correspondence between the sensory and motor hierarchies is supported by numerous anatomical, physiological, and pharmacological criteria, all of which suggest that descending pathways in sensory and motor systems are organised similarly [6]: they share, for example, a higher level of divergence compared with forward pathways and their synapses express a higher proportion of nonlinear NMDA receptors. Many aspects of ascending pathways are also concordant, with one rather glaring exception: the anatomy of forward corticocortical pathways in motor cortex. The absence (or recession) of layer 4 indicates a difference in functional organisation. However, this exception is readily interpretable – demanded even – by active inference: a motor intention is deterministic, being a model (or plan) of behaviour to be enacted, rather than a model awaiting revision by prediction errors, as in a sensory system. Prediction error generated within the motor periphery activates spinal reflex arcs, and the resulting muscle activity quashes prediction error, thereby fulfilling the prediction 73, 74. Hence, the forward pathway, through which proprioceptive prediction error ascends, is redundant [6]. Indeed, under active inference, M1 has to be shielded from the spinal proprioceptive prediction errors that engage reflexes; otherwise, it would infer that the intended movement was not being executed. In the somatosensory and exteroceptive domain, this shielding takes the form of sensory attenuation, namely, attending away from the sensory consequences of self made acts [75].

This is not to assert that there is no ascending proprioceptive prediction error (reafference discharge). Motor neurons are known to respond to proprioceptive stimulation, and so-called ‘transcortical’ reflex arcs form an important component of motor control 76, 77, 78: the corticospinal component of this loop makes direct contact with spinal motor neurons and may mediate precision (as we argue below). The fact that ascending proprioceptive predictions are computationally redundant in M1 suggests that they have been eliminated (by activity dependent plasticity) during neurodevelopment or, indeed, epigenetically. Notably, Brodmann himself observed that the inner granular layer (layer 4) is clearly formed in area 4 of the human foetus at 8 months [3]. An abnormal persistence of layer 4 has been linked to severe motor impairment in infants with cerebral palsy [79]. The supposition would be that the postnatal recession of layer 4 reflects the neurodevelopmental acquisition of motor skills: as prediction errors come to be efficiently eliminated in the periphery, their ascension to M1 is increasingly redundant and subversive. By contrast, the same prediction error relayed to S1 subserves perceptual inference, updating expectations of the proprioceptive and kinematic state of the body and, through subsequent transmission to motor cortex, helping to ensure motor intentions are fulfilled as intended [6].

## Mirror neurons

To illustrate the explanatory power of predictive coding, we now turn to the convergence of visual and motor function in mirror neurons, building on existing neuronally plausible simulations 73, 74. Originally discovered in premotor area F5, and subsequently in anterior parietal cortex [80], the key property of these neurons is to activate when performing and observing the same action [81]. They can be regarded as the apex of a visual pathway encoding action understanding 81, 82, 83 that ascends to premotor area F5 from superior temporal areas sensitive to biological motion, via parietal areas known to be engaged by visually guided reaching and grasping [84]. By their nature, mirror neurons encode motor intentions, whether enacted by oneself or used to explain the observed behaviour of others. From the standpoint of active inference, mirror neurons represent a visuomotor construct that predicts both visual and proprioceptive consequences [85]. However, how can these modes (action and action observation) operate separately? To put it bluntly: why is an observed action not, excepting the echopraxia of young infants [86], automatically mimicked?

**Figure 4:**
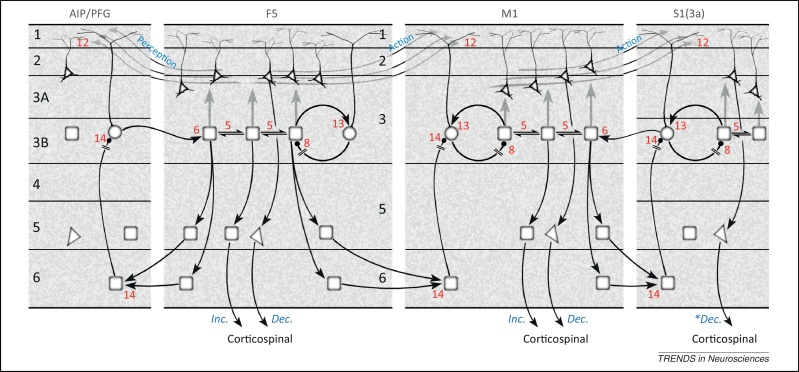
A generalised predictive coding interpretation of the circuitry sustaining visual activation of mirror neurons across four sectors of somatomotor cortex: anterior parietal (areas AIP and PFG), premotor (area F5), motor (M1), and somatosensory S1 (area 3a). Visual ‘mirror’ activation is transmitted to F5 from AIP and/or PFG, whose intrinsic and reciprocal connections are similar to those of sensory areas shown in Figure 3; AIP/PFG matching the lower level (i-1) and F5 the middle level (i). Therefore, the visual ‘mirror’ activity propagates through expectation and error units of F5, including the corticospinal expectation output from layer 5. Deep layer expectation units in area F5 also terminate as backward connections in the deep layers of M1. This can be likened to the backward connection from upper level (i+1) to middle level (i) in Figure 3. The subsequent circuitry, an internal relay from layer 6 to layer 3, reproduces that shown for sensory cortex, but this remains uncertain for motor cortex 88, 89. However relayed, the descending prediction suppresses activity of superficial error units in M1, resulting in disinhibition of superficial expectation units. Hence, layer 3 expectation units in M1 can also demonstrate visual ‘mirror’ activity that in turn is relayed to layer 5 to increment corticospinal transmission during action observation (output labelled ‘inc.’). The effectiveness of corticospinal expectations in giving rise to action is governed by activity of superficial units in F5 mediating precision. We envisage two varieties, labelled ‘perception’ and ‘action’, with complementary levels of activity. When attending to the (visual) consequences of another's action, a high level of expected precision is predicted in areas AIP–PFG, where it enhances the gain of the error units relaying visual signals to F5; a corresponding decrement in ‘action’ precision is transmitted to M1, and is also relayed corticospinally via layer 5 of both F5 and M1 (output labelled ‘Dec.’), suppressing action. Conversely, during action *per se*, enhanced corticospinal action-precision signals descending from layer 5 facilitate prediction error-dependent reflexes in the spinal cord. Thus, F5 and M1 are shown to have similar circuitry for corticospinal transmission, capable of generating mixed mirror neuron activity during action observation 87, 93, both incremental (expectation) and decremental (precision). A similar interplay is proposed for M1 and S1, whereby the superficial conveyed action-precision signal is intrinsically relayed to layer 5 of area 3a, and hypothetical corticospinal mirror neurons of the suppressive type (output labelled ‘*Dec.’).

The answer rests on separate expectations about motor and sensory precision. Effectively, proprioceptive prediction errors can be turned on during action and off during action observation. Active inference (action) occurs when peripheral proprioceptive prediction errors are afforded high precision, facilitating the spinal reflex 6, 85; conversely, the precision can be attenuated (*cf*. sensory attenuation) during action observation [85]. This attenuation does not affect action observation, because perceptual inference is driven by visual prediction errors. As noted above, precision enhances the gain of error units; in other words, corticospinal transmission of (high) precision facilitates alpha motor neurons and produces an action [6]. Figure 4 illustrates the cortical circuitry, suggested by recent physiological evidence, that could attenuate echopraxia (unintended actions) during action observation.

### Intrinsic circuitry in premotor cortex

The ascending visual signals informing an observed action reach F5 (higher area) from anterior parietal areas PFG and AIP (lower areas) [84]. The relation between F5 and PFG–AIP is analogous to that depicted in Figure 3 for a pair of higher and lower sensory areas although, due to the recession of layer 4, the ascending signals (i.e., visual prediction error) must terminate predominantly upon superficial expectation units in F5. The intrinsic circuitry of F5 shown in Figure 4 includes a new element, a descending precision signal in the corticospinal (pyramidal tract) output from layer 5. Some pyramidal tract neurons in F5 have been identified as mirror neurons that, importantly for our purposes, subdivide into two classes: one that responds to both action and action observation, according to the classic definition, and a second that is excited by action but shows suppression when observing action [87]. The latter meet our criteria for a (precision) unit whose decline in activity could attenuate spinal prediction error and so preclude echopraxia.

### Intrinsic circuitry in primary motor cortex

The descending connections from F5 to M1 and the reciprocal connections between S1 and M1 are also analogous to those illustrated in Figure 3. Intrinsic circuitry is better established in M1 than elsewhere in motor cortex. For instance, several studies have identified excitatory input to layer 5 from layers 2/3 as the most prominent internal relay 88, 89, 90. We also note previous work distinguishing three distinct classes of pyramidal neuron in the superficial layers of cat M1 on the basis of cell morphology, layer position, and orthodromic activation by stimulation of S1 91, 92: one class was monosynaptically activated from S1 (area 2), qualifying it as an expectation unit (that is driven by ascending prediction errors). The other two classes were activated at a longer (variable) latency, implying a polysynaptic pathway from S1 that would be characteristic of error and precision units, as influenced via expectation units. One of these was smaller and more superficial, consistent with our location of units mediating precision. All three displayed axon collateral branching in layer 5, although this was less extensive for the superficial class [91]. Importantly, M1 neurons can also display the mirror property 93, 94. Furthermore, some pyramidal tract neurons in M1 layer 5 have recently been identified as mirror neurons with, again, excitatory and suppressive types in regard to action observation [93], where the suppressive type is in a position to provide descending corticospinal precision (gain) control to attenuate spinal reflexes during action observation.

A subset of corticospinal neurons in M1 (known as ‘CM’ neurons) make direct contact with spinal alpha and gamma motoneurons [95], and we noted previously that the population of smaller CM neurons, also found in area 3a and likely to contact gamma motoneurons [96], would be good candidates for transmitting precision signals [6]. Therefore, we have extended the backward transmission of precision signals one step further to area 3a, with the supposition that here, too, it may be possible to find mirror neurons with a suppressive response to action observation.

### Segregated origins of descending precision signals

Clearly, it is crucial for the above scheme that neurons mediating (proprioceptive) precision that project from F5 to M1 should be anatomically distinct from those providing (exteroceptive) precision control to parietal cortex (cf. attentional control). It is commonly found that descending axons innervate multiple targets 6, 50, 51. One might predict that top-down precision control to areas AIP and PFG can be mediated by bifurcating axons, but that a separate population of F5 precision neurons projects to M1. Activity in these two systems should be complementary: when attending to one's own intentions, high motor precision in F5 would be necessary to facilitate action, whereas high sensory precision in PFG–AIP would be necessary to attend to the action of another (Box 2, Box 3).

Box 2 **Predictions**

To consolidate the requisite features of a cortical architecture performing predictive coding in the manner outlined here, we list some testable predictions:

• Pyramidal neurons belong to one of three separate classes [expectation (*Exp*), error (*Err*) or precision (*Prc*) neurons], with distinct connectional, morphological, and physiological characteristics.
• *Err* neurons are the source of forward connections, and *Exp* and *Prc* neurons the source of backward connections; there should be few, if any, neurons with axons bifurcating to both forward and backward targets, in either the superficial or deep layers.
• The minimal forward relay through an area should be disynaptic: input to *Exp*, and *Exp* to *Err* (output). Hence, ascending axon terminals should contact *Exp* neurons, but not *Err* neurons. By contrast, the minimal backward relay might be monosynaptic, for *Prc* neurons relaying through layer 2/3A, for instance.
• Pyramidal neurons with ascending extrinsic output (i.e., *Err* neurons) should have apical dendritic arborisation in layer 1, to undergo modulation by descending precision signals.
• Physiologically, *Exp* and *Err* neurons should be characterised by inverse effects of expectation. For instance, a predictable trajectory of rightward motion should suppress rightward direction-selective *Err* neurons, but enhance rightward direction-selective *Exp* neurons. There is also the possibility of ‘crossover’, that is, the rightward trajectory might exert the opposite effects upon leftward direction-selective *Err* and *Exp* neurons.
• Arising from our analysis of mirror neurons in motor cortex, we predict that: (i) mirror neurons with a suppressive response to action observation occur in layer 5 of area 3a of S1; and (ii) separate populations of *Prc* neurons in premotor area F5 are the source of backward connections to area M1, on the one hand, and to anterior parietal areas AIP and PFG, on the other.

Box 3 **Outstanding questions**

• What is the origin of precision or gain control? Or, in other words, how does the cortical microcircuit compute the expected precision of, or confidence in, sensory signals? Precision may have several separate origins. It could arise and be signalled directly through interaction between expectation and error units. Or, if explicitly represented in separate neuronal populations (as we have illustrated in this review) are there sources of precision local to sensory cortex, and/or in external regulatory systems, relating to the attentional optimisation of precision [32]?
• To what extent is precision mediated by descending cortical, and corticospinal connections, as opposed to neuromodulatory effects (e.g., as mediated by cholinergic and adrenergic transmission) acting through nonspecific neuronal terminations?
• How should lateral connections be interpreted? Anatomically, lateral connections appear to be a superposition of the forward and backward laminar patterns [5]. A literal functional interpretation would require laterally interconnected areas to be computing two distinct sets of *causes*, distinguished by a mutually reversed hierarchical order. An alternative is to consider computations relating to *states* that are not hierarchically ordered, but may still make use of interareal communication. If we envisage the expectation, prediction error and precision units for states to occupy the same layers as their equivalent neurons for causes, a symmetrical exchange of signals relating to states could easily generate broad laminar distributions of origins and terminations, as observed for the ‘intermediate’ laminar pattern of lateral connections.
• Do superficial expectation neurons ever make extrinsic connections? The population of superficial pyramidal neurons that lack extrinsic axonal projections would be classified as expectation units, but it is also plausible that other expectation neurons do make extrinsic (not necessarily backward) connections to report expectations to other brain systems in the form of corollary discharge: for example, visual expectation neurons reporting to language and/or speech cortex.
• If expectation, error, and precision units are valid categories of pyramidal neuron, they should map to existing physiological characterisations of distinct classes of sensory and motor units. In relation to the distinction between (visual) ‘simple’ and ‘complex’ neurons of V1, for instance, one might associate simple cells with expectations and complex cells with error units, at least insofar as complex cells process input from simple cells to generate ascending output, whereas simple cells provide most of the descending (corticogeniculate) output. The challenge here is to develop diagnostic criteria for physiological characteristics that identify the computational role of specific neuronal populations; for example, their characteristic frequency responses [36].

## Concluding remarks

The principle that cortical areas serving different functions should have different architectures seemed logical to Campbell and Brodmann and their successors, but few have attempted to consider how function dictates structure. Admittedly, architectural differentiation can be subtle. However, even one of the most marked architectural subtypes, agranular motor cortex, has so far resisted explanation. Predictive coding can account for the basic asymmetries of hierarchical connectivity in the cortex and has been applied here to provide a framework for laminar connectivity and a principled rationale for the recession of the granular layer in motor cortex. Furthermore, we hope to have illustrated the consilience between structure and function under this framework, through the operation of mirror neurons, which may represent one of the hierarchically deepest forms of active inference.
